# Acute effects of partial positive allosteric GABA_A_ receptor modulation by GT-002 on psychophysiological and cognitive measures: protocol for the TOTEMS phase II trial targeting cognitive impairment associated with schizophrenia

**DOI:** 10.3389/fpsyt.2025.1656792

**Published:** 2025-11-25

**Authors:** Thomas Hartwig Siebner, Karen Sandø Ambrosen, Cecilie Koldbæk Lemvigh, Christine Natasha Ryan, Mikkel Erlang Sørensen, María Hernández-Lorca, Birte Yding Glenthøj, Kit Melissa Larsen, Michael-Robin Witt, Bob Oranje, Bjørn Hylsebeck Ebdrup

**Affiliations:** 1 Center for Neuropsychiatric Schizophrenia Research (CNSR), Mental Health Center, Glostrup, Copenhagen University Hospital, Mental Health Services CPH, Copenhagen, Denmark; 2 Gabather AB, Södertälje, Sweden; 3 Department of Clinical Medicine, Faculty of Health and Medical Sciences, University of Copenhagen, Copenhagen, Denmark; 4 Danish Research Centre for Magnetic Resonance, Department of Radiology and Nuclear Medicine, Copenhagen University Hospital - Amager and Hvidovre, Copenhagen, Denmark

**Keywords:** schizophrenia, schizophrenia spectrum disorders, cognitive impairment associated with schizophrenia (CIAS), cognitive impairment (CI), hypofrontality, GABA, GABA receptor A, positive allosteric modulator (PAM)

## Abstract

**Background:**

Cognitive impairment remains a critical unmet treatment need in schizophrenia spectrum disorders (SSD). Disruption of cortical excitation/inhibition balance, involving dysfunction of the gamma-aminobutyric acid (GABA) system, leads to aberrant gamma oscillations and impaired brain network function. This disruption may manifest as hypofrontality, which is associated with deficits in basic information processing thought to underlie the cognitive impairments observed in SSD. GT-002 is a novel GABA_A_ receptor partial positive allosteric modulator. Preclinical rodent studies have demonstrated GT-002’s potential to reduce hypofrontality, while three Phase I trials have established its safety and tolerability in healthy participants.

**Aim:**

The TOTEMS Phase II trial examines acute effects of a single oral dose of GT-002 on psychophysiological measures of early information processing, including event-related electroencephalography (EEG), electromyography, and resting-state EEG in SSD patients.

**Methods:**

A single-center, double-blind, placebo- and active comparator-controlled, randomized, four-way crossover challenge trial. We will recruit 20 clinically stable patients with SSD and 30 healthy controls. Participants will receive a single dose of GT-002 (1 mg and 2 mg, developed by Gabather AB), oxazepam (15 mg), and placebo across four study drug exposure days, separated by a washout period ≥7 days. Psychophysiological measures and cognitive assessments, including the Trail Making Test and selected subtests from the Brief Assessment of Cognition in Schizophrenia and Cambridge Neuropsychological Test Automated Battery, will be conducted following each administration.

**Anticipated results:**

We hypothesize that GT-002 will improve prepulse inhibition of the startle reflex in patients relative to placebo and oxazepam, reflecting improved sensorimotor gating. Secondary hypotheses include improved mismatch negativity, selective attention, 40-Hz auditory steady-state response, and normalized resting-state EEG in SSD patients following GT-002. Exploratory endpoints include safety and tolerability of GT-002 as well as differential effects on cognition compared to oxazepam, particularly in processing speed, attention, reaction time, and working memory.

**Perspectives:**

TOTEMS is the first trial to investigate acute effects of GABA_A_ receptor modulation by GT-002 on early information processing in SSD. If successful, it will support further clinical trials of longer-term GT-002 treatment as a novel pharmacological approach to target impairments in information processing in SSD, potentially ameliorating cognitive impairments.

**Clinical trial registration:**

EU CT number 2024-519389-28-00.

## 1 Introduction

Schizophrenia spectrum disorders (SSD) are severe and debilitating mental illnesses associated with substantial impairments in real-world functioning and markedly reduced quality of life (1–3). Deficits in basic information processing are thought to underlie cognitive impairments, which represent one of the core dimensions of SSD (4, 5) and has been linked to both real-world functional impairment (6–9), and lower quality of life (10). This has led the international scientific community to propose the term ‘Cognitive Impairment Associated with Schizophrenia’ (CIAS) (11). At present, no pharmacological agent has received regulatory approval specifically for the treatment of CIAS, nor is any medication currently recommended to improve CIAS in any international guidelines (12–16). Nevertheless, multiple compounds have been investigated or are undergoing evaluation for their potential efficacy in ameliorating CIAS (17, 18), targeting diverse neurobiological mechanisms that reflect the multifactorial etiology of CIAS. However, the treatment remains complex as many different neurobiological mechanisms likely underly CIAS, such as hypofrontality, excitatory and inhibitory (E/I) imbalance at a cortical level, altered neuronal functioning and neurotransmission, grey matter volume reduction and aberrant neural network organization (19–22).

Several neurotransmitter systems and neural circuits, including the gamma aminobutyric acid (GABA), glutamatergic, dopaminergic and muscarinic pathways, converge on the regulation of E/I balance within cortical circuits (5). In these cortical circuits, the excitatory output of cortical pyramidal cells is regulated by inhibition from GABAergic interneurons (5, 20). This finely tuned balance between excitatory glutamatergic pyramidal cells and inhibitory GABAergic interneurons generates synchronized neural oscillations. Particularly, the neural oscillations occurring at approximately 40 Hz, termed ‘gamma oscillations’, seem essential to the generation of slow fluctuations in neural activity, as observed with functional magnetic resonance imaging (fMRI), that underlie functional brain networks (23, 24). In healthy individuals, these neural oscillations and functional networks have been associated with various cognitive functions, including working memory (25, 26). Multimodal evidence indicates that this E/I balance is disrupted in schizophrenia, thereby resulting in aberrant gamma oscillations that lead to brain network dysfunction (5, 20) and may manifest as hypofrontality, reflected by reduced glucose metabolism and cerebral blood flow in the prefrontal cortex (19, 27, 28). In turn, hypofrontality has been associated with disturbances in basic information processing in schizophrenia, and both factors are thought to play a role in the observed cognitive impairments (19, 27–31), although the precise causal relationships remain incompletely understood. Notably, hypofrontality is not ameliorated by treatment with the currently available antipsychotics and may even be exacerbated by such treatment (28). Several studies have indicated that antagonists of N-methyl-D-aspartate (NMDA) receptors, such as phencyclidine (PCP), ketamine and dizocilpine (MK-801) induce states of hypofrontality (27, 28, 32). This induced hypofrontality is likely underlying the schizophrenia-like deficits in electrophysiological parameters of early information processing observed in our previous studies on the effects of ketamine in healthy controls. Specifically, we found that ketamine reduced the P300 amplitude as well as processing negativity (PN) and mismatch negativity (MMN) all of which are event-related potentials (ERPs) (33, 34). When the results from both studies were combined, we also observed reductions in sensory and sensorimotor gating (35, 36). Moreover, we demonstrated deficits in sensorimotor gating, as measured by prepulse inhibition of the startle reflex (PPI), in drug-naive first-episode patients with schizophrenia (37).

Psychophysiological measures, including electroencephalography (EEG) and electromyography (EMG), are widely used to quantify neural mechanisms underlying early information processing. Examples include sensorimotor gating by PPI (38), pre-attentive sensory discrimination by MMN (39), and selective attention (SA) by the P300 amplitude (40). Patients with schizophrenia show abnormalities in all three indices compared to healthy controls (38–40), whether treated with antipsychotics (41–44) or not (37, 41, 45–50). We previously showed that the electrophysiological phenomena are related to several higher-order cognitive functions, e.g., strategy formation, visual short-term memory, verbal fluency, and cognitive inhibition and flexibility (51). In spectral analysis of resting-state EEG recordings, schizophrenia is characterized by increased delta and theta activity (52), which has been associated with dysfunctional processing of sensory input (53). Additionally, patients with schizophrenia exhibit abnormalities in the alpha frequency band (52), indicative of the above-mentioned hypofrontality (54, 55). Abnormalities are also observed in the gamma frequency band (56), involved in neuronal synchronization in both local and large-scale neuronal networks underlying a large range of perceptual and higher-order cognitive functions commonly disrupted in schizophrenia (57–59).

Studies in rodents and in humans support the involvement of the GABAergic system in the regulation of sensorimotor gating, as measured by PPI (60–64), although the dopaminergic, serotonergic, and glutamatergic systems are also involved (65). Pre-attentive auditory processing, as indexed by MMN, is thought to reflect glutamatergic NMDA receptor function and E/I balance (66, 67). Rowland et al. further provided *in vivo* evidence supporting glutamatergic and GABAergic regulation of MMN and verbal working memory function in schizophrenia (67). The 40-Hz auditory steady-state response (ASSR) provides a noninvasive measure of the ability to generate neural synchrony in the gamma range within the auditory system. Emerging evidence suggests that GABAergic neurotransmission modulates 40-Hz ASSRs and is a sensitive marker for E/I balance alterations (68–70). Several studies have investigated 40-Hz ASSRs in patients with schizophrenia, with the majority reporting 40-Hz ASSR deficits with medium-level effect sizes (69, 71, 72). In resting-state EEG of healthy participants, the benzodiazepine oxazepam is known to reduce the power of low-frequency waves, i.e., delta, theta, and alpha bands (73–76), while increasing the activity in the higher frequency ranges, i.e., beta band (73, 74, 77). The latter effect was statistically significant in as few as five participants when a single dose of 30 mg oxazepam was administered (77). Besides this activity on resting-state EEG, oxazepam is also known to reduce amplitudes of ERPs, especially of the P300 amplitude (78, 79).

Dysfunction and/or loss of parvalbumin-positive GABAergic interneurons has been proposed to disrupt the E/I balance and contribute to a diminished capacity for the gamma-frequency synchronized neuronal activity in schizophrenia (5, 71, 80–83). Accordingly, GABAergic inhibitory neurons have been suggested as potential therapeutic targets for cognitive deficits (84). The GABA_A_ receptors represent the most prevalent subtype in the central nervous system and regulate circuit activity through distinct modes of inhibition based on their localization (84–86). GABA_A_ receptors are highly expressed on postsynaptic neuronal membranes opposite to GABA releasing presynaptic nerve terminals but are also present extrasynaptically along the dendritic membrane. Synaptic GABA_A_ receptors respond to high concentrations of synaptically released GABA and mediate fast, short-lasting phasic inhibition. This form of inhibition provides timing-based signaling that defines the temporal window for neuronal network firing, thereby playing a key role in the generation and regulation of gamma or theta oscillations, as well as in maintaining network synchrony. In contrast, extrasynaptic GABA_A_ receptors respond to consistent, low concentrations of GABA and mediate slow, long-lasting tonic inhibition, which regulates neuronal excitability by modulating the amplitude and duration of postsynaptic excitatory potentials (84–86).

The GABA_A_ receptors are heteropentameric, ligand-gated chloride (Cl^−^) channels composed of different subunits that form a ring around a central ion-conducting pore in the membrane (84–86). The subunits of the GABA_A_ receptor family, encoded by 19 known genes, include α1-6, β1-3, γ1-3, δ, ϵ, π, θ, and ρ1-3. GABA_A_ receptors are generally composed of two α subunits, two β subunits, and either a γ2 or δ subunit, where the vast majority are believed to constitute of three receptor subtypes (α1β2/3γ2, α2β2/3γ2 and α3β2/3γ2) (84–86). The precise subunit composition is a major determinant of the functional characteristics of the receptor, including sensitivity to GABA, conductance, desensitisation, spatiotemporal distributions, and sensitivity to allosteric modulator (84–86).

**Figure 1:**
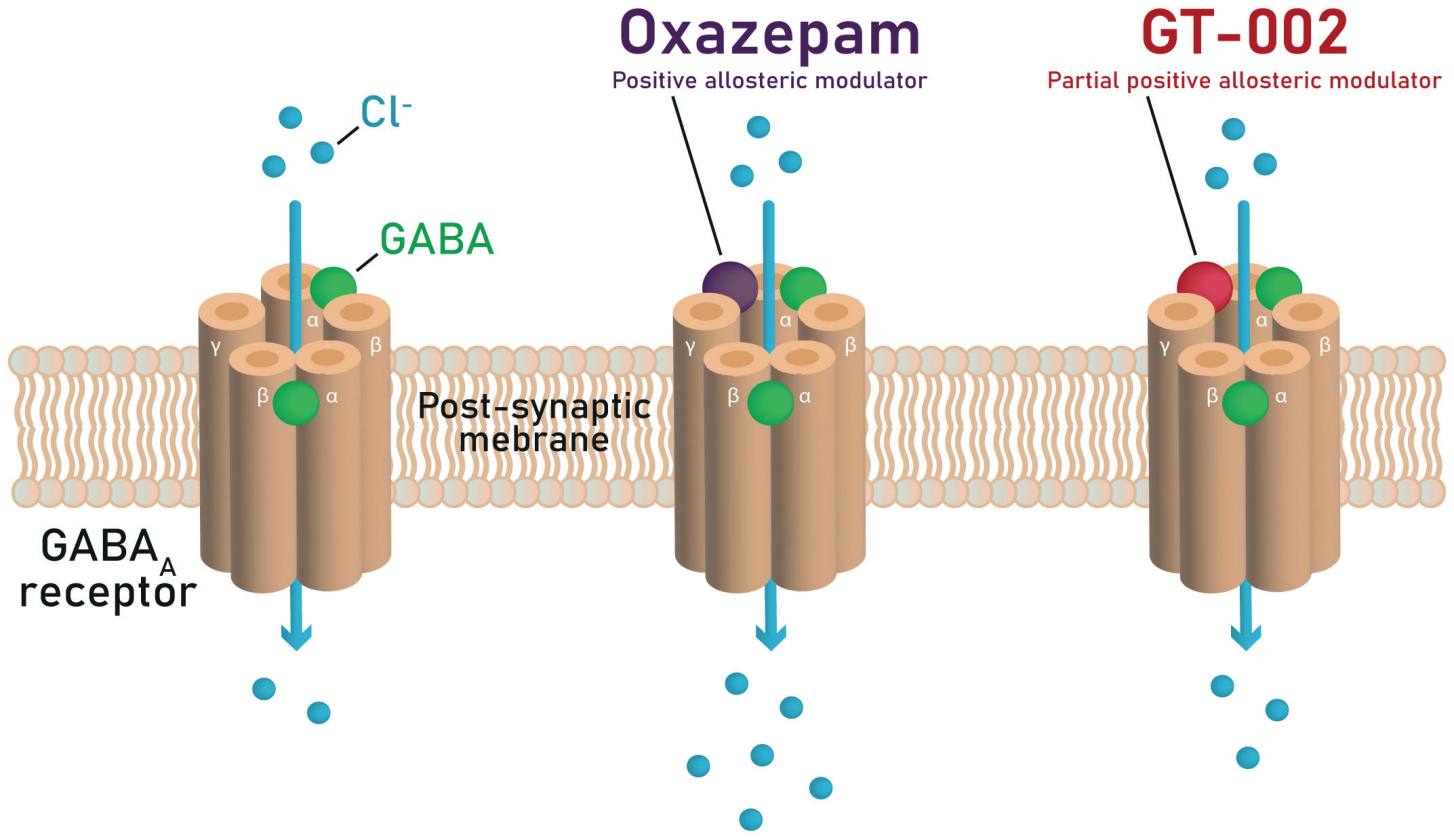
Mechanisms of GABA potentiation by oxazepam and GT-002 at the GABA_A_ receptor, illustrating that GT-002 induces only a minor enhancement of GABA-elicited Cl^−^ currents compared to oxazepam.

GABA binds to the GABA_A_ receptors at the interface between the α and β subunits, enhancing Cl^−^ conductance across the membrane (**Figure 1**). This leads to hyperpolarization of the postsynaptic membrane, hence reducing the probability that postsynaptic neurons will generate an action potential.

Benzodiazepines such as diazepam and oxazepam are non-selective positive allosteric modulators (PAMs) of GABA_A_ receptors (84, 86, 87). They enhance GABA’s action at GABA_A_ receptors by interacting with the allosteric modulatory benzodiazepine binding site formed by one of the α subunits (α1–3 or α5) and the γ2 subunit (**Figure 1**). This binding increases the frequency of the Cl^-^ channel opening in the presence of GABA, thereby increasing Cl^-^ conductance across the neuronal cell membrane and enhancing inhibitory neurotransmission. It has been suggested that benzodiazepines can mediate different effects depending upon the GABA_A_ receptor subtype in distinct neuronal circuits targeted (87–91). For example, the sedative and anterograde amnestic actions of benzodiazepines are thought to be mediated by α1-containing GABA_A_ receptors (87, 88), the anxiolytic activity by α2-containing GABA_A_ receptors (89), and the muscle relaxant activity by α2, α3, and α5 containing GABA_A_ receptors (90, 91).

Several GABA_A_ receptor modulators with diverse subunit selectivity profiles, mechanisms of action, and therapeutic indications are currently in development, as recently summarized by Thompson et al. (86). Most of these agents target epilepsy, anxiety, or depression, with relatively few explicitly targeting CIAS. GT-002 distinguishes itself within this landscape as a partial PAM at the GABA_A_ receptor, designed to potentiate GABAergic transmission to a limited extent while minimizing sedation, thereby providing a mechanistically novel approach to CIAS. Beyond GABAergic dysfunction, multiple neurotransmitter systems and pathways may be implicated, including glutamatergic, cholinergic, dopaminergic, and inflammatory mechanisms. Reflecting this complexity, compounds with distinct mechanisms are under investigation for CIAS, such as positive allosteric modulation of the α7 nicotinic acetylcholine receptor (e.g., galantamine), inhibition of d-amino acid oxidase (e.g., luvadaxistat), a combination of muscarinic agonism at M1, M4, and M5 receptors with peripheral muscarinic antagonism (e.g., xanomeline-trospium), anti-inflammatory agents (e.g., minocycline, N-acetylcysteine), trace amine-associated receptor 1 agonism (e.g., ulotaront), and selective glycine transporter 1 inhibition (e.g., iclepertin) (17, 18). Collectively, these efforts illustrate the breadth of mechanisms being explored to address CIAS and underscore the novelty of GT-002’s approach within the GABAergic domain.

The TOTEMS trial investigates the acute effects of GT-002, a novel GABA_A_ receptor partial PAM that produces minimal potentiation of GABA-elicited Cl^−^ currents, as described in the Investigator’s Brochure (IB), distinguishing it pharmacodynamically from traditional benzodiazepines (**Figure 1**). This limited potentiation may underlie the absence of sedation typically associated with benzodiazepines, while preserving GABAergic enhancement, thereby offering potential for clinical applications. The trial employs a single-dose design to assess whether GT-002 produces measurable effects on EEG and EMG without inducing sedation. Our chosen measures represent sensitive, well-established markers of early sensory and sensorimotor information processing and provide electrophysiological indicators to assess an individual’s level of hypofrontality. Acute changes in these measures may indicate engagement of neural circuits underlying hypofrontality, providing an initial signal of potential therapeutic effects. From an ethical perspective, initiating a single-dose design in this first patient trial is appropriate, as it allows preliminary pharmacodynamic evaluation while minimizing exposure. While this single-dose trial does not directly target cognitive impairments in patients with SSD, measurable effects on EEG and EMG following single dosing would provide necessary preliminary pharmacodynamic evidence to justify subsequent repeated-dose trials aimed at evaluating potential improvements in CIAS or broader cognitive function.

## 2 Methods and analysis

### 2.1 Objective of the trial

The overall objective of the TOTEMS clinical trial is to investigate the acute effects of partial GABA_A_ receptor modulation by GT-002 on psychophysiological measures, including event-related EEG and EMG, as well as resting-state EEG, in patients with SSD. Collectively, these measures provide sensitive and well-established markers of early sensory and sensorimotor information processing deficits, which are central to the pathophysiology of SSD, and serve as electrophysiological proxies for evaluating GT-002’s effect on hypofrontality. The trial endpoints are described in detail in **Table 1**.

**Table 1:** Description of the endpoints of the trial.

Primary Endpoint:
Change in PPI in patients with SSD following exposure to GT-002, placebo, or oxazepam. The primary analysis will assess the difference between 2 mg GT-002 and placebo.
Secondary Endpoints:
Changes in MMN amplitude, P300 amplitude and PN of the SA, power and phase coherence of the 40-Hz ASSR, as well as changes in EEG resting-state frequency bands in patients with SSD following exposure to GT-002, oxazepam, or placebo.
Exploratory Endpoints:
i. Safety and tolerability in both antipsychotic-treated patients with SSD and healthy controls, as measured by reported adverse events (AEs) and the visual analog mood scale (VAMS). ii. Changes in the EEG paradigms due to the differential acute effects between GT-002, oxazepam, and placebo in healthy controls. iii. Changes in cognition, including processing speed, attention, reaction time, and working memory, due to oxazepam compared to GT-002 and placebo in both patients with SSD and healthy controls. iv. The impact of antipsychotic medication type and its duration, as well as sex, age, diagnosis, and duration of illness on the acute effect of GT-002 on the EEG paradigms in patients with SSD.

### 2.2 Trial population

We aim to recruit a total of 50 participants aged 18–45 years, distributed as 30 healthy individuals with no current or past mental disorders or severe physical conditions, and 20 patients diagnosed with SSD without severe physical conditions. We aim for a balanced distribution of age and sex in both groups. Healthy participants will be recruited through advertisements. Patients will primarily be recruited through outpatient clinics in the Capital Region of Denmark and Region Zealand.

Detailed inclusion and exclusion criteria for participants are described in **Table 2**, and rules for concomitant treatments and medications before and during the trial are presented in **Table 3**.

**Table 2:** Description of the inclusion and exclusion criteria.

General inclusion criteria (for all participants)
Legally competent Males or non-pregnant, non-lactating females aged between 18 and 45 years
Additional inclusion criteria for healthy controls
No current or previous diagnosed mental disorder No first-degree relative with known major psychiatric disorder (ICD-10: F1x; F2x; F3x), defined as having received medical treatment for and/or hospitalizations related to these diagnoses
Additional inclusion criteria for patients with SSD
Fulfilling the diagnostic criteria for schizophrenia, persistent delusional disorder, acute and transient psychotic disorders, induced delusional disorders, schizoaffective disorders, other non-organic psychotic disorders, or unspecified non-organic psychosis (ICD-10: F20.x; F22.x; F23.x; F24.x; F25.x; F28; F29), prioritizing patients with a shorter antipsychotic history Treated with the same antipsychotic monotherapy for at least the last three months, including pro re nata (PRN) antipsychotic medication, and prioritizing patients treated specifically with dopamine receptor partial agonists, irrespective of formulation Clinically stable for a minimum of the last three months, i.e., without hospitalizations for SSD or recently intensified psychiatric care

General exclusion criteria (for all participants)
Prior serious adverse reaction, hypersensitivity, or intolerance to benzodiazepines, GT-002, placebo, or their excipients Ongoing treatment with benzodiazepines Severe (co-morbid) physical condition Pregnancy Lactation Unwillingness or inability to use contraception methods during the study period and until the end of the relevant systemic exposure period *(women of childbearing potential only)* Hearing impairment compromising the planned EEG assessments. Physical or language impairments that negatively impact the accuracy of cognitive assessment data or verified mental retardation (IQ ≤ 70) Clinically relevant findings on physical examination at the screening visit Clinically relevant abnormalities on 12-lead ECG at the screening visit Clinically relevant findings in laboratory samples at screening Participation in a clinical study involving study medical treatment administration within three months prior to screening or in more than 2 clinical studies within 1 year prior to the screening visit Positive results from urine drug tests Unwillingness to refrain from donating blood or blood products during the study
Additional exclusion criteria for healthy controls
Lifetime substance dependence (ICD-10: F1x.2) (exception: nicotine dependence, F17.x) or any use of illicit drugs within the 12 months prior to inclusion Any prescribed medications and over-the-counter medications (exceptions specified in **Table 3**) within 3 weeks prior to the first study drug administration
Additional exclusion criteria for patients with SSD
Current substance dependence (ICD-10 F1x.2) (exception: nicotine dependence, F17.x) or any use of illicit drugs within the three months prior to inclusion Any previous or current coercive measure as per Danish legislation Electroconvulsive therapy (ECT) in the last three months

**Table 3:** Rules for concomitant treatments and medications before and during the trial.

To ensure participant safety, prevent potential interactions with the investigational drugs, and avoid confounding effects on outcome measures, all concomitant medications and relevant medical history will be thoroughly assessed during screening. Based on the type of medication, dose, and half-life, the TOTEMS investigators will determine whether a medication may be continued, paused, or should result in exclusion from the trial.
All participants:
The use of any medication that may interfere with either the study drugs or influence trial endpoints is prohibited *from one month prior to the first study drug exposure day and throughout trial participation*. Prohibited medications include: • GABAergic agents, including barbiturates, benzodiazepines, and Z-drugs • GABA transaminase inhibitors • GABA reuptake inhibitors • GABA antagonists • GABA dietary supplement • Regular use of sleeping pills (excluding melatonin) • Opioids • Antiepileptics • Antidepressants • Muscle relaxants • Antihistamines • Anticholinergic agents • Moderate or strong inducers or inhibitors of CYP3A4 *From three weeks prior to the first study drug exposure day until the first safety follow-up*, all participants are encouraged to refrain from or minimize use of any concomitant medications. The following exceptions apply: • Paracetamol and ibuprofen for PRN use (excluding depot formulations), provided they are not taken within 24 hours prior to study drug exposure days • Vitamin D supplements, provided they are not taken within 24 hours prior to study drug exposure days • Hormonal contraception, permitted throughout the study, provided it was initiated at least one month prior to the first study drug exposure day
Additional criteria for patients with SSD
At screening, patients must have been on a stable regimen of antipsychotic monotherapy for *at least three months*, defined as: • No dose increases during this period • A maximum dose reduction of 50% in daily dose One additional antipsychotic may be prescribed PRN (as needed) but must not be taken within 24 hours prior to study drug exposure days. If permanent changes in antipsychotic medication occur during the trial, the participant will be excluded at the discretion of the TOTEMS investigators.

### 2.3 Study design

The TOTEMS clinical Phase II trial follows a single-center, double-blind, placebo- and active-comparator-controlled, randomized four-way crossover design with single exposure.

**Figure 2:**
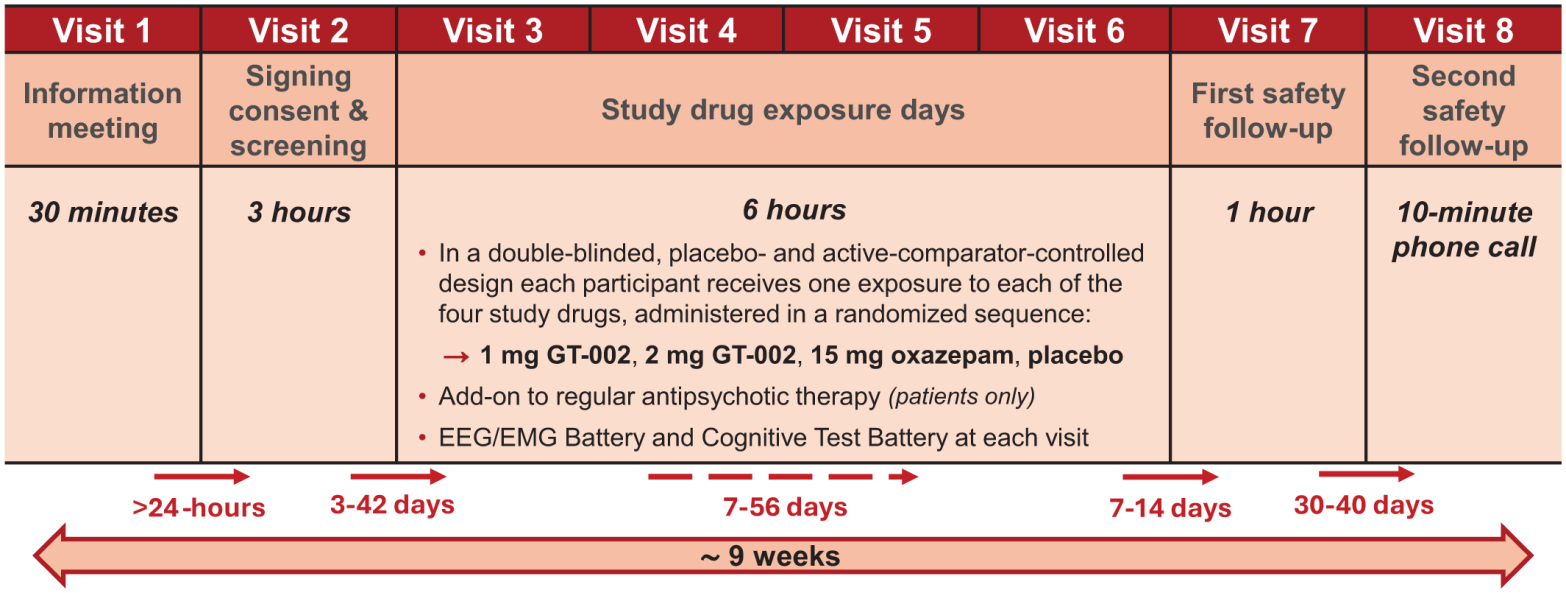
Overview of the eight visits conducted during the TOTEMS clinical trial, with time intervals indicated between each visit.

Each participant will attend up to 8 visits at the Center for Neuropsychiatric Schizophrenia Research (CNSR), as outlined in **Figure 2**.

The visits include an information session, a screening visit (after which participants will be scheduled for the first study drug exposure within 3 to 42 days), followed by four study drug exposure days (each separated by a washout period of at least 7 days and no more than 56 days between them), and two final follow-ups. The first safety follow-up examination will take place 7 to 14 days after the last study drug exposure day (Visit 6), followed by a second safety follow-up telephone call 30–40 days after the first safety follow-up or study discontinuation.

**Figure 3:**
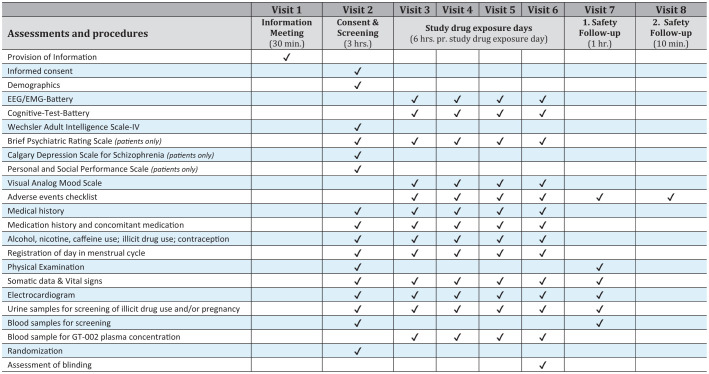
Overview of assessments and study procedures performed by each visit.

**Figure 4:**
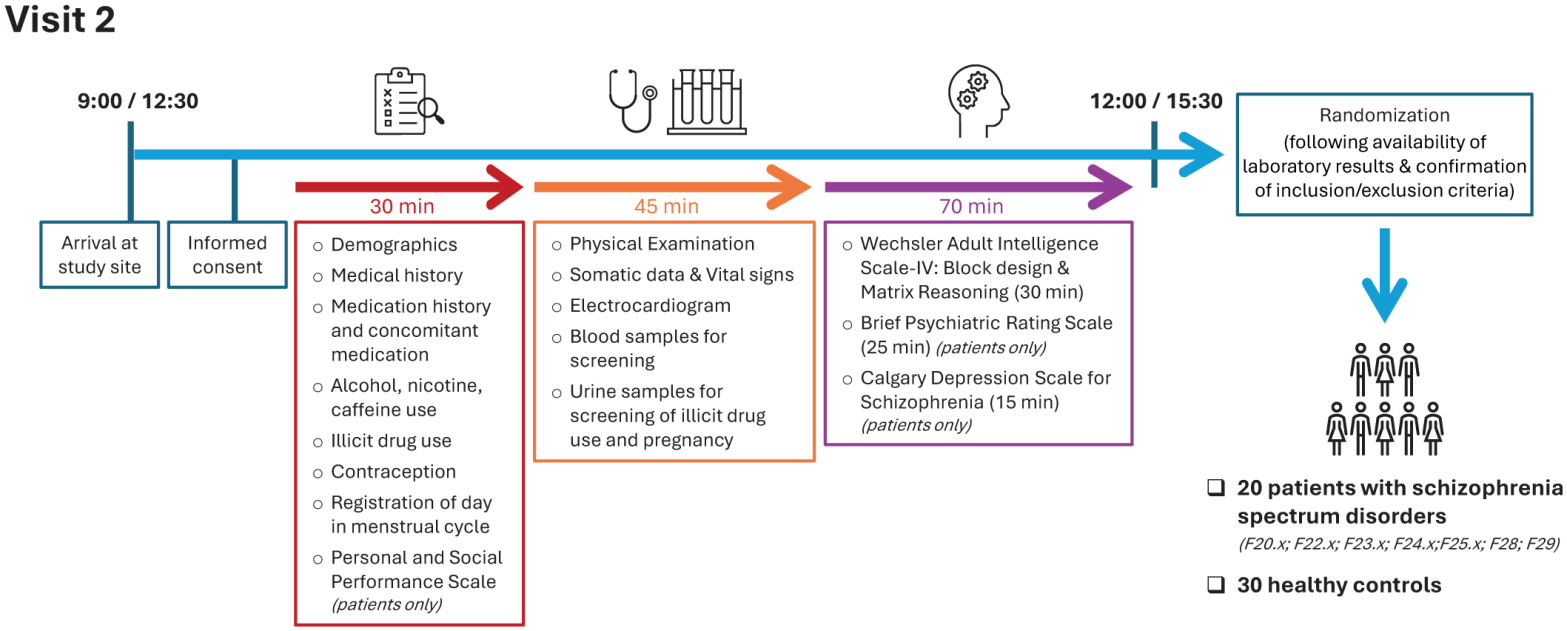
Timeline of Visit 2. Diagnostic codes include schizophrenia (ICD-10: F20.0), persistent delusional disorder (F22.x), acute and transient psychotic disorders (F23.x), induced delusional disorders (F24.x), schizoaffective disorders (F25.x), other non-organic psychotic disorders (F28), and unspecified non-organic psychosis (F29).

**Figure 5:**
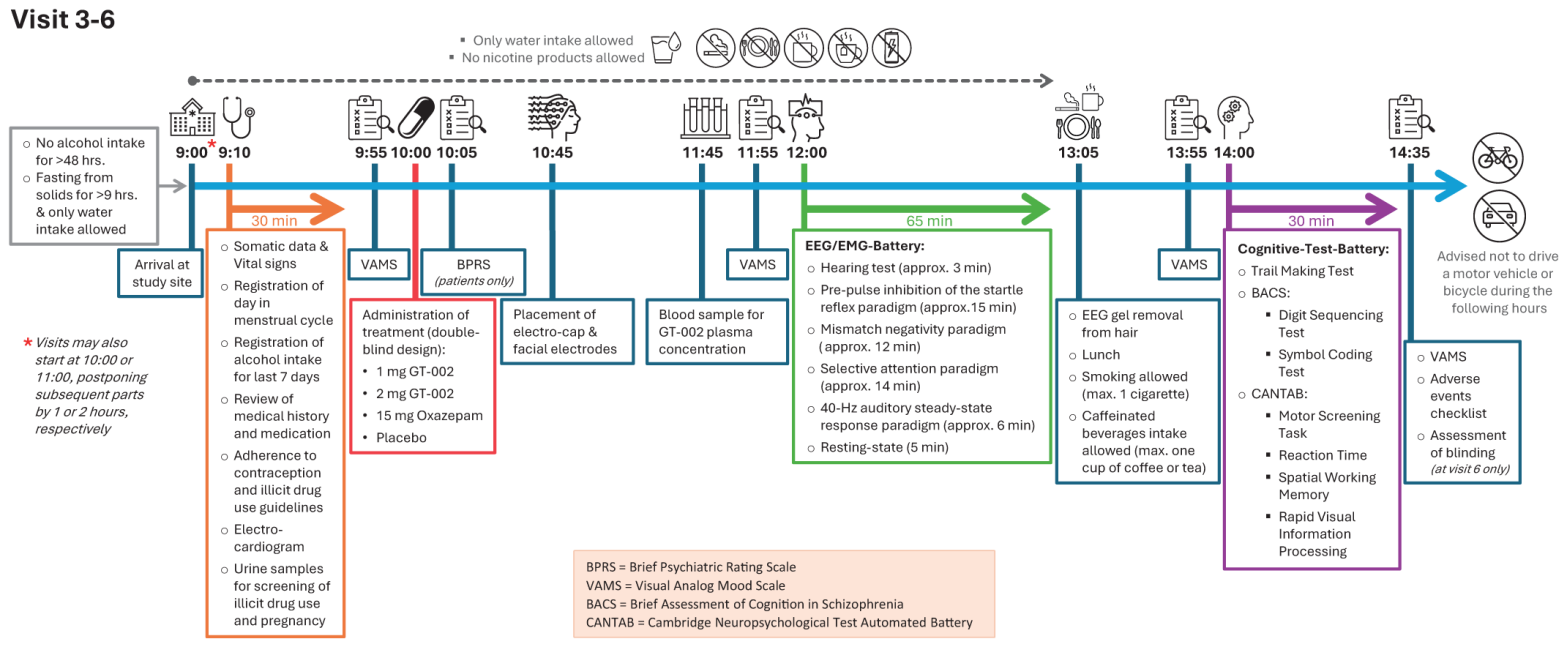
Timeline of Visits 3-6.

**Figure 6:**
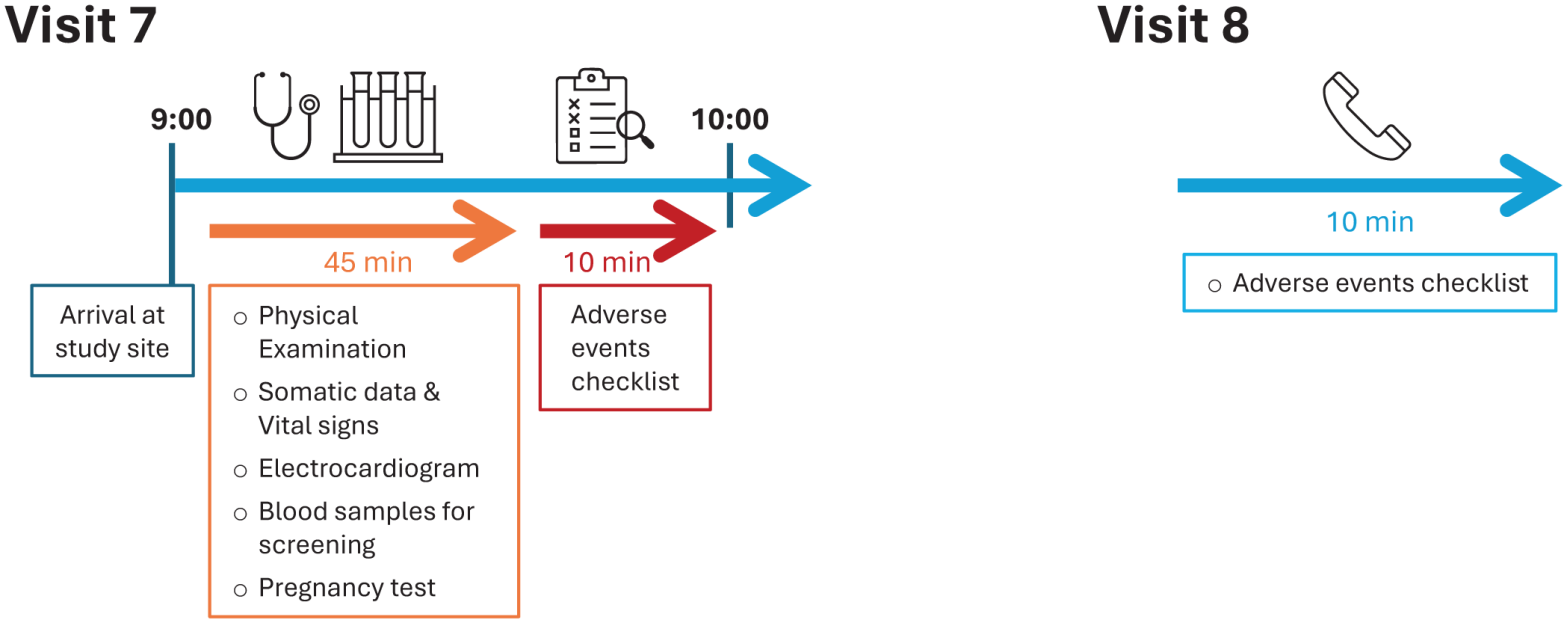
Timeline of Visits 7 and 8.

We aim to schedule the first study drug exposure day following the availability of laboratory results from screening, as well as ensuring minimal intervals between subsequent study drug exposure days. The 56-day upper limit between study drug exposure days accommodates scheduling constraints, including holidays and participant availability. Consequently, we anticipate each trial subject’s participation to extend for a minimum of 9 weeks, with a theoretical maximum duration of 38 weeks. For a detailed description of each visit see **Figures 3**–**6**.

Data collection is scheduled to occur over a period of 27 months from the start of the trial.

Participants will receive a compensation of DKK 3,000 (≈ € 400) upon completion of the full study. If a participant chooses to withdraw before completion, reimbursement will cover only the visits completed. Patients will be provided with taxi transport to and from the study site for Visits 2-7, with the cost covered by the study. Alternatively, if patients prefer to use public transportation, these expenses will also be compensated.

### 2.4 Randomization and blinding procedure

Randomization of study drugs will be performed centrally at the clinical research unit of the Capital Region Pharmacy of Denmark and will be conducted separately for healthy participants and patients.

With four different study drug exposures and each participant receiving each study drug once, 24 possible administration sequences exist for randomization. The randomization codes for the study drug exposures will not be accessible to the TOTEMS investigators until data analysis for each group is complete.

Unblinding will occur in two phases: first, after all data from the healthy participants has been analyzed, and second, after all data from the patients has been analyzed. This approach accommodates the expected longer recruitment period for patients while maintaining blinding integrity. To ensure further objectivity in the data analysis, all data processing will be conducted automatically with batch jobs. This approach eliminates any potential subjectivity in the statistical interpretation or the scoring of the data.

Due to the potential alteration of GT-002’s pharmacokinetics, the GT-002 capsule cannot be encapsulated and differs significantly from oxazepam tablets. Additionally, producing a placebo identical to the 15 mg oxazepam tablet is not feasible given the formulation characteristics. Therefore, two types of placebos are required for this trial: the first placebo (for GT-002) is a soft gelatine capsule that is identical to the 1 mg GT-002 capsule, and the second (for oxazepam) is an encapsulated placebo tablet that is identical to the encapsulated oxazepam tablet. Both placebos will contain no active drug substance.

**Figure 7:**
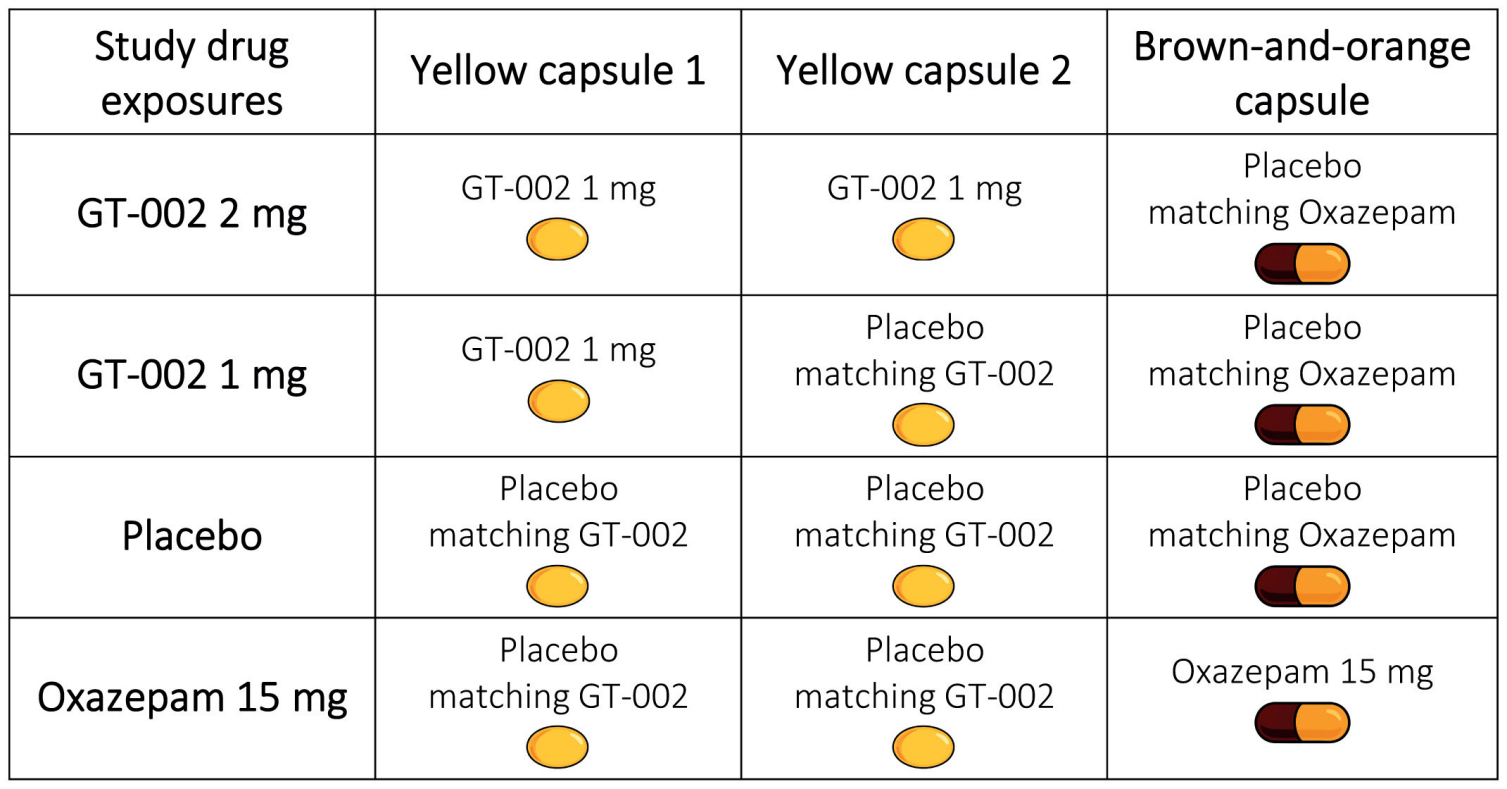
Composition of study drug exposures, each comprising of three capsules administered per exposure day, according to the randomized sequence assigned to each participant.

As GT-002 is supplied in 1 mg capsules, the 2 mg dose will be administered as two capsules. To ensure double blinding, each study drug exposure will include three capsules, as outlined in **Figure 7**: two yellow capsules (either GT-002 or matching placebo) and one brown-and-orange capsule (either encapsulated oxazepam tablet or matching encapsulated placebo tablet).

Gabather will supply GT-002 and its matching placebos, while the Capital Region Pharmacy of Denmark will provide oxazepam tablets (15 mg, Alternova), the corresponding placebo tablets, and the capsules used to encapsulate both.

### 2.5 Study drugs

#### 2.5.1 GT-002

The preclinical and clinical data on GT-002 are based on the IB for GT-002 (Edition No. 4, dated 20 December 2024), as supplied by Gabather AB. GT-002 is a novel, orally administered drug candidate that targets the GABA_A_ receptor and acts as a partial PAM. It is a selective α3-preferring PAM with limited α5 modulation, as demonstrated in electrophysiological patch-clamp studies (data not included in the IB), suggesting a unique pharmacological profile and potential translational value in clinical trials. *In vitro* competitive binding studies demonstrated dose-dependent displacement of the GABA_A_ agonist radioligand [^3^H]-muscimol, while no displacement of the dopamine D_3_ antagonist ([^3^H]-methylspiperone) or D_5_ antagonist ([^3^H]-SCH 23390) radioligands was observed. Further characterization using radioligand competition binding assays showed that GT-002 also displaced [^3^H]-flunitrazepam, which binds to the benzodiazepine site. Thus, GT-002 demonstrated high-affinity binding to this site (K_i_ = 0.57 nM) and was ten times more potent than diazepam, with an IC_50_ of 0.68 nM compared to 7 nM for diazepam. Receptor binding selectivity profiling using an *in vitro* radioligand binding assay revealed that GT-002 did not significantly affect any of the selected panel of receptors (i.e. dopamine, glutamate, GABA, glycine, and serotonin) and transporters (i.e. GABA transporter). However, potential off-target interactions revealed that, aside from the GABA_A_ receptor, GT-002 had significant effects on the dopamine transporter (DAT) at 1 μM and 10 μM but not at 0.1 μM. Since there is an ~800-fold difference in the IC_50_ values between DAT and the GABA_A_ receptor (0.55 μM vs. 0.68 nM, respectively), significant pharmacological interaction with DAT is not expected. Thus, the risk of off-target dopaminergic effects is considered minimal. Ion channel profiling demonstrated that, in contrast to diazepam, GT-002 elicited only minor potentiation of GABA-elicited Cl^−^ currents, as illustrated in **Figure 1**. GT-002 induced only 10-20% of the ion channel activation compared to diazepam, consistent with a profile of partial positive allosteric modulation at the GABA_A_ receptor. Preclinical efficacy studies have demonstrated significant effects of GT-002 in animal models of schizophrenia using NMDA antagonists PCP and MK-801. GT-002 demonstrated beneficial effects on cognition, memory, and social interaction, as assessed by the Social Interaction Test and the Novel Object Recognition Test.

In three clinical trials in healthy volunteers, including a first-in-human single ascending dose study, a multiple ascending dose study, and an EEG/fMRI target engagement study, GT-002 was safe and well-tolerated with no serious adverse events reported (92, 93). No drug-related changes in cognitive function or mood were observed. There are no known contraindications to its administration. Pharmacokinetic analysis indicated that GT-002 reached peak plasma concentration (T_max_) approximately 2 hours post-administration, had an elimination half-life (T_1/2_) of around 20 hours, and was not associated with sedative effects. In the TOTEMS trial, participants will receive 1 mg and 2 mg doses, both within the established safe and well-tolerated range. For further details regarding the three clinical trials in healthy volunteers, see the **Supplementary Material**.

#### 2.5.2 Oxazepam

Oxazepam (ATC-code: N05BA04) is an authorized medicinal product with extensive clinical use and a well-established safety profile, approved by the Danish Medicines Agency. For this trial, it will be sourced from the manufacturer Alternova and used as an active comparator to GT-002, selected for its GABA_A_ receptor targeting and pharmacokinetic similarity. Oxazepam is approved for the treatment of anxiety and agitation but is used off-label in the TOTEMS trial as a tool compound. Participants will receive a single 15 mg dose, which is the minimum recommended by both the manufacturer and the Danish Medicines Agency. The tablets cannot be accurately divided to achieve a lower dose (e.g., 7.5 mg), as the scored line on the tablet is intended solely to facilitate swallowing rather than to provide precise fractional dosing. Administering an imprecise lower dose could introduce variability in oxazepam’s pharmacodynamic effects, which is why a 15 mg dose was selected for the trial. While sedation is a known effect of oxazepam, this characteristic is deliberately utilized as part of its role as an active comparator, enabling differentiation between a classical benzodiazepine profile with sedation and the intended non-sedating profile of GT-002.

All participants will be screened for contraindications to oxazepam. Exclusion criteria reflect those listed in the official summary of product characteristics, including hypersensitivity, myasthenia gravis, sleep apnea, severe hepatic impairment, and acute respiratory depression. Only individuals in overall good physical health will be enrolled, and relevant parameters will be monitored throughout the study.

### 2.6 Safety measures

The described inclusion and exclusion criteria (**Table 2**) will exclude those at higher risk for toxicities from the experimental compounds. Moreover, participants will undergo safety monitoring during the study, including assessment of the nature, frequency, and severity of adverse events (AEs). Safety monitoring will include on-site medical supervision during study drug administration days, with a physician physically present to manage any adverse events. Participants will have access to study personnel during working hours and emergency contact options outside office hours. Two safety follow-up visits will be conducted after completion of all four study drug exposures. An AE checklist, covering 46 symptoms across multiple organ systems, will be used at the end of each study drug exposure day and during follow-up visits to ensure comprehensive monitoring. The checklist includes common and compound-specific adverse events associated with both oxazepam and GT-002. Suspected unexpected serious adverse reaction (SUSAR) will be reported to the Danish Medicines Agency in accordance with applicable regulatory timelines. All patients will be clinically stable at enrollment and continue antipsychotic monotherapy throughout the study.

In case of medical emergency or SUSAR, the participant’s treatment sequence can be unblinded. Sealed envelopes with unblinding codes will be stored securely at the study site. The decision to unblind will be at the discretion of the TOTEMS investigators. Unblinding may also occur if required by local laws or regulations.

Women of childbearing potential must use highly effective contraception (failure rate <1% per year) during the study drug exposure period and for five days after the last dose, based on the elimination half-life of GT-002 (approximately 20 hours).

All procedures will be conducted with attention to participant comfort and well-being. Participants may withdraw at any time without reason.

### 2.7 Electroencephalography and electromyography

The EEG/EMG battery comprises the PPI paradigm, MMN paradigm, SA paradigm, 40-Hz ASSR paradigm, and resting-state. See a detailed description of the paradigms in **Table 4**. These paradigms were selected based on evidence demonstrating moderate-to-large group differences between patients with schizophrenia and healthy controls (71, 72, 94, 95), with the MMN, 40-Hz ASSR, and resting-state EEG additionally demonstrating good test–retest reliability across sites and feasibility for standardized, automated data acquisition (96). The EEG/EMG battery, followed by the cognitive test battery, will be conducted 2 hours after dosing, corresponding to the Tmax of both GT-002 and oxazepam. EEG recordings will be conducted using BioSemi^®^ hardware (BioSemi, Netherlands) with a cap containing 64 Active Two electrodes, arranged according to the extended 10–20 system. All auditory stimuli will be presented by a computer running Presentation^®^ software (Version 24.0, Neurobehavioral Systems, Inc., Berkeley, CA, USA), and delivered binaurally through stereo insert earphones (E-A-RTONE™ GOLD 3A Insert Earphones, 3M United Kingdom PLC, Bracknell, UK). The eye-blink component of the acoustic startle response in the PPI paradigm will be measured by recording EMG activity from the right orbicularis oculi muscle. For this purpose, two electrodes will be placed under the right eye for PPI and habituation assessment. The first of these will be aligned with the pupil, while the other will be positioned laterally in the direction of the outer canthus of the eye. The EMG recordings will also be assessed using BioSemi^®^ hardware.

**Table 4:** Detailed description of the paradigms in the EEG/EMG battery.

Hearing test (approximately 3 min)
Participants are screened for hearing deficits with pure tones of 500, 1000, and 6000 hertz (Hz) at a sound intensity of 40 decibel (dB) (duration: 40 ms). The tones are presented randomized across both ears (three times for each ear, totaling up to 18 trials). Intertrial intervals are randomized between 5 and 10 seconds. Participants are asked to push one of two buttons corresponding to the left or right side of stimulation.

PPI paradigm (approximately 15 min)
Following a 1-minute acclimatization period to 70 dB background white noise, three stimulus blocks are presented against the same background. Blocks 1 and 3, which are identical, assess habituation via 8 pulse-alone trials (115 dB, 20 ms white noise bursts) with randomized intertrial intervals of 10–20 s. Block 2 evaluates percentage PPI through a randomized sequence of pulse-alone and prepulse-pulse trials. Prepulses (20 ms white noise bursts at 76 or 85 dB) precede the pulse stimulus (as in blocks 1 and 3) with stimulus onset asynchronies of 60 or 120 ms. Ten trials of each prepulse condition are presented, totaling 50 trials, with intertrial intervals randomized between 10–20 s.

MMN paradigm (approximately 12 min)
The MMN paradigm comprises 1800 binaurally presented auditory stimuli. Four stimulus types are used: standard tones (1000 Hz, 50 ms; 82%) and three types of deviants, consisting of frequency deviant (1200 Hz, 50 ms; 6%), duration deviant (1000 Hz, 100 ms; 6%), and double deviant (1200 Hz, 100 ms; 6%). All stimuli are presented at 60 dB in a single run, with interstimulus intervals randomized between 400–500 ms. Participants are instructed to ignore the sounds and focus on a nature documentary featuring animals and landscapes presented on the screen.

SA paradigm (approximately 14 min)
The auditory SA task consists of 400 stimuli in total, randomly presented to either ear, comprising standard tones (80%) and deviant tones (20%) differing in pitch (1000 Hz vs. 1200 Hz). Stimuli (60 dB, 50 ms) are delivered with interstimulus intervals of 700–900 ms. Participants are asked to press a button in response to deviants in a designated ear, with ear assignment counterbalanced across participants. After the first task, attention shifts to the opposite ear. Attended deviants are not presented consecutively. The task yields ERP components (N100, P200, P300), PN, and behavioral measures (hits, misses, false alarms, reaction time).

40-Hz ASSR paradigm (approximately 6 min)
To elicit steady-state 40 Hz activity, participants are presented binaurally with 1 ms auditory clicks at a constant 40 Hz repetition rate (25 ms inter-click interval). Each 1-second click train is followed by a 2-second pause, yielding a 3-second stimulus onset asynchrony. A total of 120 trials are delivered at 85 dB.

Resting-state (5 min)
Resting-state EEG is recorded for 5 minutes with eyes open, during which the participants are instructed to think of no particular topic.

During EEG/EMG recording, participants will be seated in a comfortable chair and instructed to maintain their gaze on a fixation cross positioned at eye level on a screen directly opposite from their seating position, approximately 2.5 meters away. The only exception will be the MMN paradigm, during which participants will be presented a muted nature documentary featuring animals and landscapes on the screen. The total duration of the EEG/EMG battery is approximately 70 minutes.

EEG and EMG data from each paradigm will undergo preprocessing prior to analysis. Preprocessing steps will generally include filtering (high- and low-pass), epoching, and artifact rejection. Artifact detection and rejection procedures may vary between paradigms and will be performed using established software tools, such as BESA^®^, Python, or MATLAB, according to the specific requirements of each analysis. The preprocessing pipeline will follow methodologies applied in our previous publications [e.g., Rydkjaer et al., 2020 (46); Bak et al., 2017 (51); Oranje et al., 2017 (49); Randau et al., 2019 (50); Larsen et al., 2018 (97)], ensuring consistency and reproducibility. Detailed preprocessing parameters, including filter settings, epoch lengths, and artifact rejection criteria, will be reported in the methods section of subsequent publications to ensure reproducibility and transparency.

### 2.8 Cognitive assessment

The cognitive tests have been selected based on literature demonstrating that acute benzodiazepine administration induces sedation, drowsiness, psychomotor slowing, anterograde amnesia, and impaired learning (98, 99), which represent potential effects of oxazepam at the administered dose in this trial. A single 2 mg oral dose of lorazepam (which has a similar T_max_ and a slightly longer T_1/2_ compared to oxazepam) has been shown to significantly impair immediate recognition, reaction time, and delayed memory (100). Cognitive side effects have also been reported across multiple domains, including processing speed and memory, following the same dose (101). Moreover, two 2 mg oral doses administered 12 hours apart adversely affected cognitive performance, including domains such as attention, working memory, verbal memory, and executive functions, among others (102).

We include the Trail Making Test and selected subtasks from the Brief Assessment of Cognition in Schizophrenia (BACS), including the Digit Sequencing Test and Symbol Coding Test, as well as tasks from the Cambridge Neuropsychological Test Automated Battery (CANTAB), including the Motor Screening Task (MOT), Reaction Time (RTI), Spatial Working Memory (SWM), and Rapid Visual Information Processing (RVP). BACS was developed for repeated measurement in clinical trials of patients with schizophrenia and is sensitive to the cognitive deficits observed in this group (103). CANTAB is a well validated computerized neuropsychological test battery that has previously been used in a variety of clinical samples including patients with schizophrenia (104, 105).

In addition, during Visit 2, we will estimate intelligence (IQ) using two subtests from the Wechsler Adult Intelligence Fourth Edition (WAIS-IV), i.e., block design and matrix reasoning, as these have shown the strongest correlation with Full Scale IQ in the Danish reference population (106).

### 2.9 Clinical assessments

The Brief Psychiatric Rating Scale (BPRS) is an 18-item rating scale that assesses psychiatric symptoms occurring over the preceding three days, using a five-point Likert scale ranging from 0 to 4 (107). The Danish translation by Anne Marie Johansen will be used (108). Given the single-dose nature of the study, significant changes in patients’ symptom severity are not anticipated. Although included patients are considered clinically stable, fluctuations in symptom severity may occur during the study. At screening, the BPRS is administered alongside the Calgary Depression Scale for Schizophrenia to establish baseline psychiatric symptom severity. During the study drug exposure days, the BPRS will be administered immediately following drug administration. Thus, no treatment-related effects are expected at this time point given the T_max_ of two hours for both GT-002 and oxazepam. Moreover, due to its retrospective assessment window of three days, the BPRS is not suited for detecting hyper-acute treatment effects, which are instead monitored using an adapted Visual Analog Mood Scale. Therefore, the BPRS is administered to patients only at screening and at each study drug exposure day to assess symptom stability across the trial, thereby providing the possibility to control for potential confounding influences of natural symptom variability on outcome measures between study drug exposure days.

The Calgary Depression Scale for Schizophrenia (CDSS) is a nine-item clinician rated outcome measure assessing symptoms experienced over the preceding two weeks and is the most widely used scale for assessing depression in schizophrenia. It has excellent psychometric properties, internal consistency, inter-rater reliability, sensitivity, specificity, and discriminant and convergent validity (109). The CDSS is administered to patients only at screening to identify the presence and severity of depressive symptoms at baseline.

The Personal and Social Performance Scale (PSP Scale) evaluates four domains: socially useful activities, personal and social relationships, self-care, and disturbing and aggressive behaviors (110, 111). Each domain is rated using a 6-point severity scale based on a structured clinical interview, resulting in a total score ranging from 1 to 100, where higher scores indicate better functioning. The PSP is administered to patients only at screening to assess social and personal functioning, and ratings are based on the participant’s functioning over the past month.

An adapted version of the Visual Analog Mood Scale (VAMS) is used to assess eight specific mood states: Afraid, Confused, Sad, Angry, Energetic, Tired, Happy, and Tense. Each mood is rated using a horizontal visual analog scale, anchored with a “neutral” descriptor at the left end and the target mood descriptor at the right end. Respondents mark the point along the scale that best represents their current emotional state. Scores range from 0 to 100, with 100 indicating the maximal intensity of the mood and 0 indicating minimal intensity or absence of that mood. The VAMS is administered to all participants repeatedly throughout each study drug exposure day (prior to study drug administration, before the EEG/EMG battery, prior to the cognitive test battery, and following completion of testing) to sensitively detect acute mood fluctuations and potential sedative effects during each study drug exposure day.

### 2.10 Patients’ involvement in the design of the trial

Following the finalization of the trial protocol, we consulted an advisory group comprised of individuals with lived experience of psychosis and their relatives, to obtain feedback, identify potential challenges, and gather suggestions to enhance the trial’s feasibility. Two panel members with SSD reviewed the protocol and provided practical recommendations, including flexible scheduling, procedures for support outside of office hours, transportation assistance through the option of taxi transport, and appropriate, ongoing monetary compensation. All suggestions were carefully considered and incorporated into the trial design.

### 2.11 Power calculation and justification of the scheduled number of participants

Since this is the first trial investigating GT-002 in patients, there are no previous data on its effects on EEG, EMG, or cognition in patients with SSD. To provide a reference point, we considered our previous study using single doses of clonidine, which demonstrated a significant improvement in PPI, a highly sensitive measure of sensorimotor gating, with a Cohen’s d of 0.73 (42). While differences in drug mechanisms and study design preclude direct extrapolation, this informed a working estimate of Cohen’s d of 0.70 for GT-002. This assumed effect size is consistent with expectations for a compound progressing from a Phase II proof-of-concept trial to a subsequent repeated-dose Phase II trial and ultimately a Phase III trial, as smaller effects would indicate insufficient clinical efficacy to warrant further development of the compound.

The power calculation, based on a paired t-test with a significance level (α) of 0.05 and a desired power of 0.80, indicates that 18 patients would be required to detect the assumed effect. To provide a safety margin, we will include 20 patients. A sensitivity analysis indicates that this sample size achieves ~84% power for d = 0.70 and ~79% for d = 0.65, suggesting that the study retains acceptable power even if the true effect size proves slightly smaller than assumed.

In addition to the patient group, we will include 30 healthy controls for exploratory analyses. Prior research indicates that a significant effect of 30 mg oxazepam on resting-state EEG can be detected with as few as 5 healthy controls (77). To ensure a robust analysis and adequately evaluate the effects of GT-002 in comparison to oxazepam, we aim for a sample size sufficient to detect a moderate-to-large effect size (d = 0.55). Based on this, 28 participants would be required, which we rounded up to 30.

### 2.12 Planned statistical analyses

The primary analysis will consist of a single planned comparison using a paired t-test to evaluate changes in PPI between 2 mg GT-002 and placebo in patients with SSD. This statistical method allows for a direct assessment of the efficacy of GT-002 relative to placebo in the primary outcome measure. As only one statistical test is performed for the primary endpoint, no adjustment for multiplicity is required. A two-sided significance level of 0.05 will be applied throughout.

Secondary analyses will primarily employ linear mixed models (LMMs) to account for the study design, including multiple drug conditions, various EEG paradigms, and time intervals between measurements. LMMs will enable a comprehensive analysis of both fixed effects (e.g., drug conditions) and random effects (e.g., individual subject variability), thereby providing a more nuanced understanding of GT-002’s impact beyond PPI. This approach will enable direct comparisons between GT-002, oxazepam, and placebo across EEG paradigms. To account for multiple testing in these secondary and exploratory analyses, multiplicity will be controlled using the Holm–Bonferroni method or similar approach.

Exploratory analyses will be conducted to evaluate the impact of factors such as antipsychotic medication type, duration, sex, age, diagnosis, and duration of illness on the acute effects of GT-002 on the EEG paradigms in patients with SSD. Additionally, the differential acute effects of GT-002, oxazepam, and placebo on EEG paradigms and cognition in healthy controls will be assessed using appropriate statistical models. Baseline analyses will be conducted to assess participant characteristics and to facilitate exploratory correlation analyses.

Across all analyses, effect sizes (e.g., Cohen’s d for paired comparisons, partial η² for linear mixed models) with 95% confidence intervals will be reported alongside p-values. Statistical analyses will be performed using R software, and model assumptions (e.g., normality of residuals) will be assessed, with non-parametric alternatives applied as appropriate.

### 2.13 Biobank

A research biobank will be established for this trial to store blood samples collected from participants for the measurement of GT-002 plasma concentrations. The samples will be stored at the study site until the end of the data collection, after which they will be transferred to Gabather AB for batch analysis and destroyed immediately following the completion of the analyses. All procedures will comply with relevant regulations, including the General Data Protection Regulation (GDPR) and the Danish Data Protection Act, ensuring ethical handling and disposal of the biological material.

### 2.14 Data management, storage, and sharing

All personal data will be handled in full compliance with the General Data Protection Regulation (GDPR) and the Danish Data Protection Act. Patient data will be entered into an electronic Case Report Form (eCRF), depersonalized using subject numbers, and treated as confidential. The project is registered in Privacy, the research registry and of the Capital Region of Denmark (Approval no.: p-2024-15354). The sponsor/principal investigator will allow authorized access to trial data and relevant documents for monitoring, auditing, and inspection by relevant authorities. Upon completion of the TOTEMS trial and following peer-reviewed publication, the full trial results will be provided to Gabather AB in pseudonymized form.

## 3 Anticipated results and discussion

The TOTEMS clinical trial will compare the acute effects of GT-002 with those of the widely used benzodiazepine oxazepam, given that both compounds act on the GABA_A_ receptor. The primary and secondary hypotheses are presented in **Table 5**. The expected differences in effect between GT-002 and oxazepam are likely attributable to GT-002’s high-affinity and selective binding to the GABA_A_ receptor, which is currently its only identified high-affinity target, and to its action as a partial PAM. This is in contrast to oxazepam, which functions as a full PAM at the same receptor.

**Table 5:** Description of the primary and secondary hypotheses for both patients with SSD and healthy controls.

Primary hypothesis:
GT-002 will improve/normalize an impaired PPI in patients with SSD, while oxazepam will reduce PPI, and placebo will have no effect on PPI.
Secondary hypotheses:
i) GT-002 will improve/normalize MMN amplitude in patients with SSD, while it will not affect PPI of healthy controls. In both patients and healthy controls, oxazepam will reduce MMN amplitude, and placebo will have no effect on MMN amplitude. i) GT-002 will improve/normalize reduced P300 amplitude and PN in patients with SSD, while it will not affect these parameters in healthy controls. In patients as well as healthy controls oxazepam will reduce P300 amplitude and PN, and placebo will have no effect. ii) Both GT-002 and oxazepam will alleviate the reduction in power and attenuation in phase coherence of the 40-Hz ASSR in patients with SSD. However, GT-002 will assume an intermediate position between oxazepam and placebo. In healthy controls, both GT-002 and oxazepam will increase the power of the 40-Hz ASSR. iii) In both patients and healthy controls, GT-002 will increase alpha band power in resting-state EEG. Oxazepam will increase activity in higher frequency bands (Beta: 13–26 Hz) and reduce the activity of low-frequency waves (by reducing power in Delta (0.5–3 Hz), Theta (3.5–7 Hz), and Alpha bands (10.5–13 Hz). Placebo will have no impact on resting-state EEG. iv) In both patients and healthy controls, oxazepam will impair processing speed, attention, reaction time, and working memory, while GT-002’s acute effects on these cognitive domains will be at the level of placebo.

Schizophrenia spectrum disorders are characterized by considerable neurobiological and cognitive heterogeneity, which poses challenges for detecting pharmacodynamic effects. While enrichment based on specific baseline abnormalities (e.g., PPI deficits or cognitive impairment) could increase sensitivity to particular mechanistic effects, such an approach entails several limitations. First, selectively including patients with abnormal baseline values introduces a risk of regression to the mean, whereby extreme measurements naturally drift toward average levels upon retesting. This could potentially exaggerate apparent treatment effects or mask adverse pharmacodynamic responses. Second, enrichment may introduce a sample bias and reduce external validity, thereby limiting generalizability and potentially obscuring how the compound acts across the broader clinical population.

Evidence from meta-analytic and large multicenter studies supports the presence of robust neurophysiological abnormalities in schizophrenia. Meta-analyses of sensorimotor gating evaluated by PPI [San-Martín et al., 2020 (94)], MMN [Umbricht and Krljes, 2005 (95)], and 40-Hz ASSR [Thuné et al., 2016 (71); Zouaoui et al., 2023 (72)] consistently demonstrate moderate-to-large impairments in patients with schizophrenia compared with healthy controls. These findings were further corroborated by a large multicenter, industry-led study using standardized EEG acquisition and automated analysis pipelines [Cecchi et al., 2023 (96)], which confirmed that patients with schizophrenia exhibit deficits in MMN and 40-Hz ASSR consistent with prior literature. Collectively, this evidence indicates that neurophysiological deficits are prevalent and reliably measurable at the group level in patients with schizophrenia.

In this proof-of-concept trial, we will include the full schizophrenia spectrum, rather than only patients with schizophrenia, to capture the range of cognitive and neurophysiological variability relevant to GABAergic dysfunction, ensuring generalizability and allowing assessment of the overall pharmacodynamic effects of GT-002 in a clinically representative sample. As this is the first trial in a patient population, our main objective is to evaluate how GT-002 modulates early information processing across the full spectrum of schizophrenia-related neurophysiological variability, rather than focusing solely on individuals with marked baseline deficits. The crossover design helps account for interindividual variability, while the inclusion of healthy controls provides an external benchmark for interpreting pharmacodynamic effects. Exploratory analyses will examine whether baseline biomarker or cognitive measures moderate treatment response, thereby informing targeted or stratified approaches in subsequent confirmatory trials.

A potential limitation of this trial is that the sedative effects of oxazepam may be noticeable to some participants. To mitigate this, oxazepam and its matching placebo are both encapsulated to ensure they are visually indistinguishable, thereby preserving blinding and maintaining the integrity of the double-blind design.

While a primary endpoint is a prerequisite in randomized controlled trials, TOTEMS also includes multiple secondary endpoints reflecting other electrophysiological parameters of early information processing. Should the primary endpoint of this Phase II trial prove negative, findings from the secondary endpoints may still justify and encourage further clinical investigation of GT-002.

Although the trial is not designed to directly address CIAS, it aims to determine whether a pharmacological signal on EEG and EMG can be detected following a single dose of GT-002. Positive acute effects would provide preliminary evidence of engagement of the neural circuits underlying hypofrontality, offering a rationale for subsequent repeated-dose trials to evaluate the therapeutic potential of GT-002 as a novel pharmacological approach for alleviating hypofrontality and improving cognitive impairments in patients with SSD. The minor risks associated with trial participation are outweighed by considerable potential future benefits, including clinically significant improvements for patients with SSD and other disorders involving deficient basic information processing. If our hypotheses are confirmed, the trial will provide initial evidence for targeting hypofrontality in schizophrenia, with potential implications for improving treatment and quality of life. Gabather and CNSR will then evaluate initiation of follow-up studies aiming to determine the effectiveness of long-term GT-002 treatment on cognition and symptomatology in patients with SSD. Should a repeated-dose Phase II trial demonstrate efficacy, this would support progression to a larger, international Phase III trial. Ultimately, if development continues to demonstrate efficacy and safety, GT-002 could become globally available, potentially benefiting patients worldwide. Its therapeutic scope may also extend to other disorders where basic information processing is compromised, such as dementia and depression.
