# Supplementary material for: Acute effects of partial positive allosteric GABAA receptor modulation by GT-002 on psychophysiological and cognitive measures: protocol for the TOTEMS phase II trial targeting cognitive impairment associated with schizophrenia
Source: Front Psychiatry. 2025 Nov 25;16:1656792. doi: 10.3389/fpsyt.2025.1656792 (PMC12746507; doi:10.3389/fpsyt.2025.1656792)
Supplement: Supplementary file 1 [file DataSheet1.pdf]

frontiers

Supplementary Material

Clinical data on GT-002

The following clinical data on GT-002 are based on the Investigator's Brochure for GT-002 (Edition No. 4, dated 20 December 2024), as supplied by Gabather AB.

In three clinical trials – a first-in-human single ascending dose (SAD) study in healthy young males, a multiple ascending dose (MAD) study, and an EEG/functional magnetic resonance imaging (fMRI) target engagement study in healthy males and females – GT-002 was safe, well-tolerated, and demonstrated a pharmacokinetic profile consistent with once-daily administration. In the SAD trial, GT-002 was administered at six different dose levels (0.05 mg, 0.1 mg, 0.3 mg, 0.5 mg, 1 mg, and 2 mg), and was found to be safe and well tolerated at doses of 0.05-2 mg. The time to peak plasma concentration (T_{max}) was around 2 hours (+/- 0.5 hours), and the maximum observed plasma concentration (C_{max}) ranged from 0.61 ng/ml at 0.05 mg to 31.48 ng/ml at 2 mg. The pharmacokinetics revealed dose-proportional plasma profiles and an elimination half-life ($T_{1/2}$) of about 20 hours. No serious adverse effects were observed. There were no clinically significant abnormal findings in the vital signs or ECG recordings. No changes indicative of an adverse reaction to administration of GT-002 were observed in the clinical chemistry or hematology safety laboratory values. No sedative drug effects or drug-related changes in cognitive function or mood were observed. Headache and nasopharyngitis were the most commonly reported treatment-emergent adverse events; however, it was concluded that no adverse events observed in the SAD trial could be attributed to GT-002 dosing.

In the MAD trial, healthy male and female subjects received doses of 0.3 mg, 1 mg, or placebo daily for 7 days. The C_{max} at 0.3 mg was 6.39 ng/ml on day 1 and 8.39 ng/ml on day 7, while at a dose of 1 mg, it was 17.4 ng/ml on day 1 and 29.47 ng/ml on day 7. The $T_{1/2}$ following administration of multiple doses ranged from about 17.44 hours (0.3 mg) to 24.39 hours (1 mg). GT-002 demonstrated dose-proportional C_{max} and $AUC_{0-\tau}$ following multiple oral doses, with no clinically relevant accumulation observed in plasma with oral once-daily doses of 0.3 mg or 1 mg. The most common treatment-emergent adverse event reported was headache, which occurred at similar rates in both the GT-002 and placebo groups, indicating it was unrelated to GT-002 dosing. There were no serious adverse events or discontinuations due to adverse events in the MAD trial. No sedative drug effects or drug-related changes in cognitive function or mood were observed following repeated treatment with GT-002.

Additionally, no drug-related changes were seen in saccadic peak velocity or body sway. No overall patterns were observed for any clinical laboratory parameter, vital signs, ECG and body weight.

The target engagement EEG/fMRI trial in 17 healthy volunteers has been completed but is still under analysis.

To summarize, no serious adverse effects were observed across all three clinical trials, and there are currently no known contraindications to the administration of GT-002. As effects on reproduction and fetal development have not been studied, GT-002 is contraindicated in pregnancy. Women of childbearing potential must use highly effective contraception (failure rate <1% per year) during the study drug exposure period and for five days after the last dose, based on the elimination half-life of GT-002.
